# 2789. Cefepime Versus Non-Cefepime/Non-Carbapenem Therapy for Blood Stream Infections Caused by Non-ESBL-Producing *Serratia Marcescens*: A Subgroup Analysis of The *Carbapenem-Serratia* Multicenter Retrospective Cohort Study

**DOI:** 10.1093/ofid/ofad500.2400

**Published:** 2023-11-27

**Authors:** Abdallah Mughrabi, Julian Maamari, Timothy Phillips, Afaq Alabbasi, Aislinn Brooks, Rinat Nuriev, Lisa Zenkin, Bertrand Jaber, Claudia Nader

**Affiliations:** St. Elizabeth's Medical Center - Boston University School of Medicine, Boston, Massachusetts; St. Elizabeth's Medical Center - Boston University School of Medicine, Boston, Massachusetts; St. Elizabeth's Medical Center, Boston, Massachusetts; St. Elizabeth's Medical Center - Boston University School of Medicine, Boston, Massachusetts; Steward St. Elizabeth's Medical Center of Boston, Rutland, Massachusetts; St. Elizabeth's Medical Center - Boston University School of Medicine, Boston, Massachusetts; St. Elizabeth's Medical Center - Boston University School of Medicine, Boston, Massachusetts; St. Elizabeth's Medical Center - Tufts School of Medicine, Boston, Massachusetts; St. Elizabeth's Medical Center - Tufts School of Medicine, Boston, Massachusetts

## Abstract

**Background:**

*Serratia Marcescens* causes serious infections. Carbapenems were prescribed due to the bacterium’s ability to develop inducible AmpC-mediated resistance. Recent IDSA guidance recommends ceftriaxone and in high-burden cases, cefepime. Evidence on the role of cefepime in the treatment of *Serratia* bacteremia is scarce. We aim to evaluate the outcomes of patients who received cefepime-containing empiric and/or definitive therapy (CFCT) to those receiving non-cefepime therapy (NCFCT).

**Methods:**

This is a sub-analysis of a retrospective study of 7 Massachusetts acute care hospitals from January 1, 2015, to October 31, 2022. We included hospitalized adults (≥18 years) with *Serratia Marcescens* bacteremia. IRB approval was obtained. Demographics, clinical course characteristics, and choice of antibiotic therapy were collected. Primary outcome was 30-day mortality, and secondary outcome was composite antimicrobial failure (see Definitions in **Table 1**). Chi Square and Mann-Whitney U tests were computed using SPSS statistical package.

**Results:**

The original *Carbapenem-Serratia study* had 73 out of 128 patients who met the inclusion criteria (**Figure 1).** The non-carbapenem group (n=56) was further studied for the purpose of this subanalysis and was divided into CFCT (n=15) and NCFCT (n=41). The two subgroups were similar in baseline comorbidities, severity measures, and sources of bacteremia **(Table 1).** Neither 30-day mortality nor the composite outcome of antimicrobial failure were significantly different with relative risks of 0.94 (95% CI 0.16 to 5.3, P=0.948) and 1.21 (95% CI 0.27 to 5.4, P=0.800), respectively.

Study exclusion flow-chart with subanlaysis.

**Figure:**
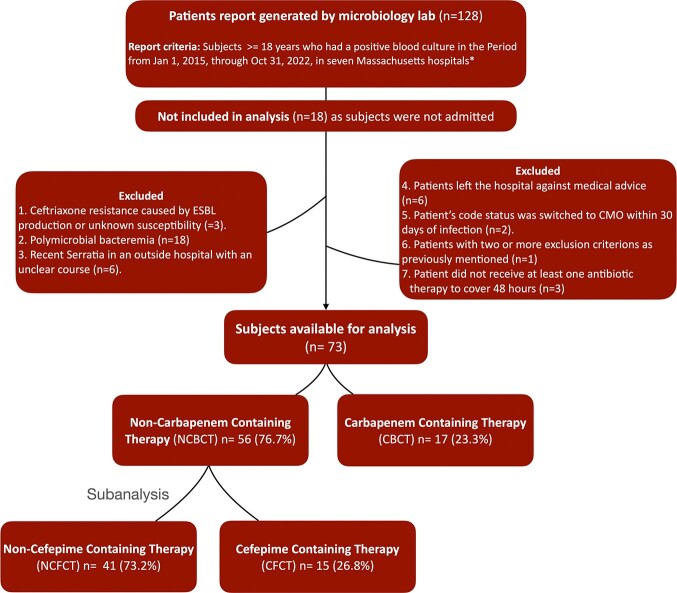

ESBL phenotypically defined by ceftriaxone resistance (MIC <1).

Characteristics and outcomes of patient with Serratia bacteremia treated with a cefepime versus non-cefepime nor carbapenem therapy regimen.

**Figure:**
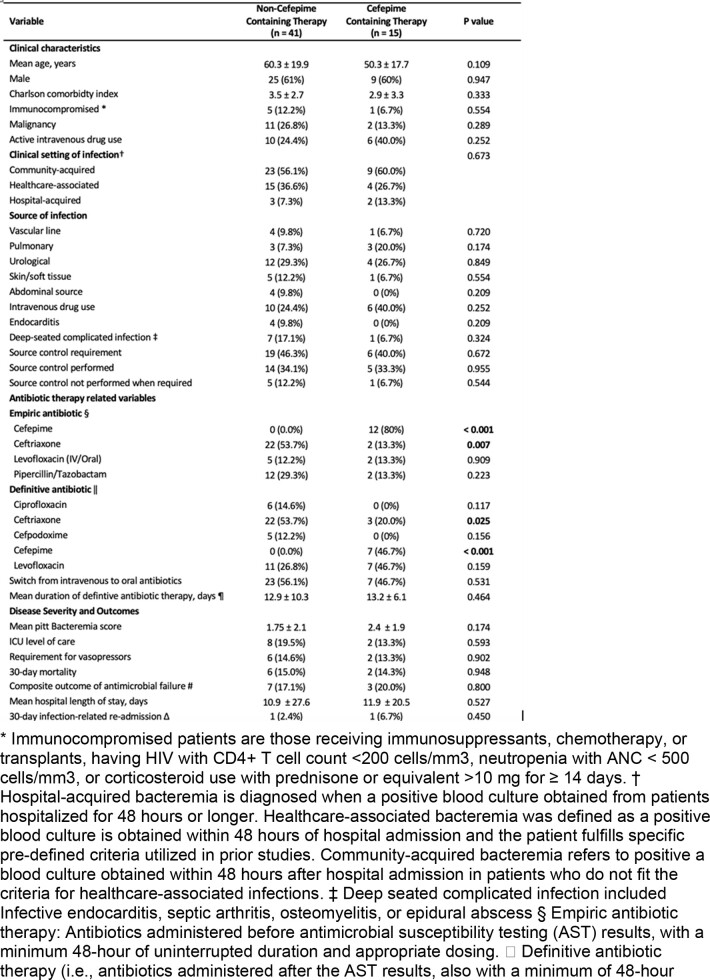

* Immunocompromised patients are those receiving immunosuppressants, chemotherapy, or transplants, having HIV with CD4+ T cell count <200 cells/mm3, neutropenia with ANC < 500 cells/mm3, or corticosteroid use with prednisone or equivalent >10 mg for ≥ 14 days. † Hospital-acquired bacteremia is diagnosed when a positive blood culture obtained from patients hospitalized for 48 hours or longer. Healthcare-associated bacteremia was defined as a positive blood culture is obtained within 48 hours of hospital admission and the patient fulfills specific pre-defined criteria utilized in prior studies. Community-acquired bacteremia refers to positive a blood culture obtained within 48 hours after hospital admission in patients who do not fit the criteria for healthcare-associated infections. ‡ Deep seated complicated infection included Infective endocarditis, septic arthritis, osteomyelitis, or epidural abscess § Empiric antibiotic therapy: Antibiotics administered before antimicrobial susceptibility testing (AST) results, with a minimum 48-hour of uninterrupted duration and appropriate dosing. || Definitive antibiotic therapy (i.e., antibiotics administered after the AST results, also with a minimum of 48-hour duration). ¶ Duration of total definitive antibiotic therapy was counted from day of AST results until last day of definitive antimicrobial therapy. # Microbiological failure, microbiological relapse, in- hospital mortality, 30-day mortality, infection-related hospital readmission, or recurrent Serratia bateremia within 30 days. Microbiological failure refers to the growth of Serratia after 48 hours of definitive antibiotic therapy, while microbiological relapse is the regrowth of Serratia after a negative blood culture (Kunz Coyne et al., 2023). Note: Two patients had recurrence of Serratia bacteremia within 30 days, neither were resistant to third generation cephalosporins. Δ Infection-related re-admission was defined when a patient is readmitted not solely for a procedure or surgery, and they experience fever, worsening leukocytosis, or required escalation of antimicrobial therapy from the definitive therapy of the index admission.

**Conclusion:**

Averting cefepime in the antimicrobial therapy of *Serratia* bacteremia is not associated with a significant increase in 30-day mortality or the composite outcome of antimicrobial failure. When applicable, a narrower beta-lactam therapy such as ceftriaxone may be appropriate. Limitations include a small sample size, with higher risk for type II error, and overlap in definitive antibiotic therapy. Further prospective studies are needed to confirm these findings, especially in patients with deep-seated infections.

**Disclosures:**

**All Authors**: No reported disclosures

